# Collision Cutaneous Neoplasms Consisting of Melanoma and Basal Cell Carcinoma

**DOI:** 10.5826/dpc.1103a37

**Published:** 2021-07-08

**Authors:** Maryam Aghighi, David Chercover, Maral Rahvar

**Affiliations:** 1 Department of Pathology, Rutgers Robert Wood Johnson Barnabas Health, Livingston, NJ, USA; 2 Department of Pathology and laboratory medicine, Lions Gate Hospital, North Vancouver, BC, Canada; 3 Department of Pathology and laboratory medicine, University of British Columbia, Vancouver, BC, Canada

**Keywords:** Lentigo Maligna Melanoma, Basal Cell carcinoma, Collision tumor

## Introduction

A Collision tumor is a rare neoplastic lesion presenting 2 histologically distinct tumors sharing the same anatomical location. Basal cell carcinomas (BCC) have been reported to collide with other skin lesions. BCC present more than other neoplasms in collision tumors and have a high prevalence among Caucasians. Collision of BCC and other benign or malignant mesenchymal, epithelial, and melanocytic tumors have been reported. Similarly, collision of melanoma may take place with other cutaneous lesions such as BCC.

Melanocytes are generally found within the basal epidermal layer and outer follicular root sheath, thus, the entrapment of non-atypical melanocytes in growing basal cell carcinomas (BCC) is not unusual as it may provide a suitable environment for the melanocytes [1].

These melanocytes are usually isolated, without cytological atypia and proliferation markers. However, the presence of atypical melanocytes within BCC is uncommon.

It has been shown that lentigo maligna and superficial spreading melanoma in situ have association with BCC. Melanoma cells may unexpectedly surround or colonize within BCC and hence mimic invasive melanoma [2].

## Case Presentation

Here we report a case of a 91-year-old male patient presenting a translucent plaque with brown areas of pigmentation on his left lateral canthus. His clinical history reported multiple BCCs, squamous cell carcinomas, and an invasive melanoma of the right cheek. The lesion was removed with curettage following clinical diagnosis of BCC.

**Figure 1:**
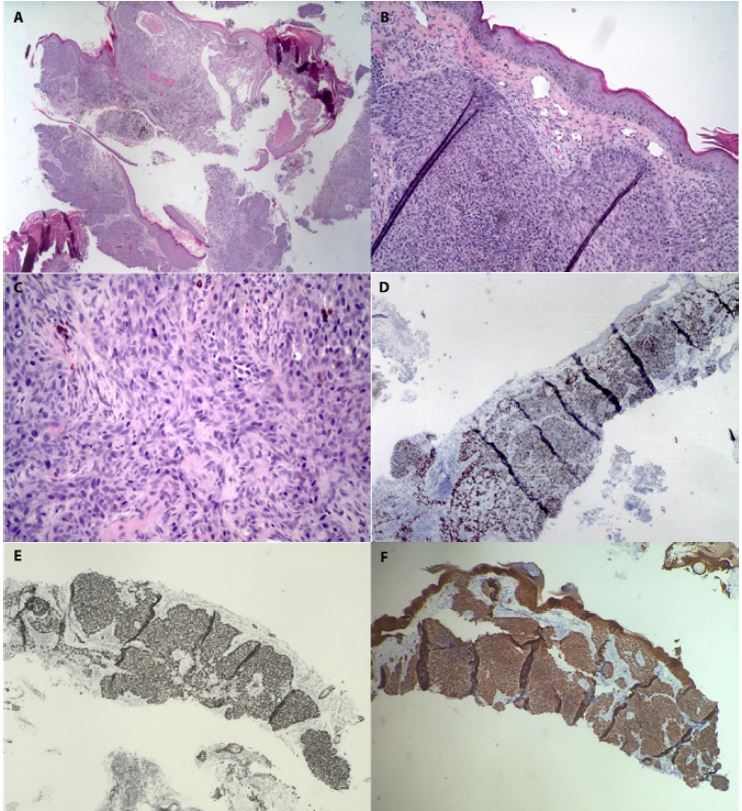
Collision of LMM and BCC. Skin presenting in-situ melanoma heavily infiltrating the BCC dermal nodules. (A) (H&E, × 20). (B) (H&E, ×100). (C) (H&E, ×200). (D) Atypical melanocytes are highlighted by SOX10 (H&E, ×20). (E) p63 marker (H&E, ×20). (F) Pan-cytokeratin (H&E, ×20).

A histologic examination showed the epidermis with a malignant melanoma in-situ overlying solid nodules of a pigmented basal cell carcinoma. Atypical melanocytes heavily percolated into dermal nodules of the BCC (Figure 1 A–C). Further immunohistochemical evaluation with melanocytic and epithelial markers (melanin A, SOX-10, p63, pan-cytokeratin) confirmed the diagnosis (Figure 1, D–F). The BCC contained about 50% atypical melanocytes. An unequivocal invasive melanoma component was not detected.

## Conclusions

Tumors composed of melanocytic and epithelial components are categorized into biphenotypic, combined, colonized, and collision tumors. Biphenotypic tumors grow from a single precursor tumor cell that differentiates into 2 distinct tumors. Combined tumors consist of 2 different, but combined, cell groups. Immunohistochemistry is useful to distinguish among the 2 groups. In colonized tumors, 1 tumor type diffuses but remains within the other tumor. Finally, collision tumors consists of 2 different adjacent tumor cells with clear distinguishable boundaries.

Different hypotheses regarding the development of these tumors have been discussed. One hypothesis suggests that collision tumors arises from 2 separate tumors in vicinity. Another hypothesis suggests that the environment of epithelial tumors changes thanks to the production of cytokines and growth factors that induce melanocytes colonization.

There are about 30 cases reported in the literature of dual cutaneous neoplasms consisting of melanoma and BCC. Challenges in the diagnosis of these dual tumors are due to a chance of misdiagnosis of melanoma as a pigmented BCC. While looking for melanocytic markers, it is better to examine the dubious/uncertain lesions by immunophenotyping. Isolated melanocytes can be seen at the border of pigmented BCC. However, atypical melanocytes in clusters may reveal the presence of melanoma or its metastasis in BCC.

Since the prognosis of the 2 entities is independent, wider excision is indicated, to exclude the possibility of an invasive malignant melanoma.
